# Tris–base buffer: a promising new inhibitor for cancer progression and metastasis

**DOI:** 10.1002/cam4.1032

**Published:** 2017-05-29

**Authors:** Arig Ibrahim‐Hashim, Dominique Abrahams, Pedro M. Enriquez‐Navas, Kim Luddy, Robert A. Gatenby, Robert J. Gillies

**Affiliations:** ^1^ Department of Cancer Imaging and Metabolism H. Lee Moffitt Cancer Center Tampa Florida; ^2^ Department of Integrated Mathematical Oncology H. Lee Moffitt Cancer Center Tampa Florida; ^3^ Department of Radiology H. Lee Moffitt Cancer Center Tampa Florida

**Keywords:** Acidosis, buffer therapy, GLUT‐1, metastasis, pancreatic cancer, prostate cancer, Tris–base

## Abstract

Neutralizing tumor external acidity with oral buffers has proven effective for the prevention and inhibition of metastasis in several cancer mouse models. Solid tumors are highly acidic as a result of high glycolysis combined with an inadequate blood supply. Our prior work has shown that sodium bicarbonate, imidazole, and free‐base (but not protonated) lysine are effective in reducing tumor progression and metastasis. However, a concern in translating these results to clinic has been the presence of counter ions and their potential undesirable side effects (e.g., hypernatremia). In this work, we investigate tris(hydroxymethyl)aminomethane, (THAM or Tris), a primary amine with no counter ion, for its effects on metastasis and progression in prostate and pancreatic cancer in vivo models using MRI and bioluminescence imaging. At an ad lib concentration of 200 mmol/L, Tris effectively inhibited metastasis in both models and furthermore led to a decrease in the expression of the major glucose transporter, GLUT‐1. Our results also showed that Tris–base buffer (pH 8.4) had no overt toxicity to C3H mice even at higher doses (400 mmol/L). In conclusion, we have developed a novel therapeutic approach to manipulate tumor extracellular pH (pHe) that could be readily adapted to a clinical trial.

## Introduction

The extracellular pH (pHe) of tumors is acidic as compared to normal tissue. Despite the fact that decreased extracellular tumor pH is associated with numerous cellular H+ exporting mechanisms, such as Na–H exchangers, vacuolar ATPases, and carbonic anhydrases 1, 2, 3, nevertheless, glycolysis is considered the major cause of tumor acidity. Although hypoxia (low oxygen) will stimulate glucose fermentation to lactate via the Pasteur effect 4, elevated lactate production is observed even in well‐oxygenated tumors, which Warburg described 70 years ago 5.

We have observed that this acidic pHe is important, and perhaps sufficient, for the transition from an in situ to an invasive cancer, and have proposed a microenvironmental model of carcinogenesis that is focused on the barriers to cellular proliferation at different stages of cancer evolution 6, 7. We have investigated this model at the microscopic level using window chambers and computer models to show that tumor acidosis promotes invasion that is enhanced by poor perfusion 8, 9. Furthermore, in many tumor types, acute or chronic treatment with low pH promotes in vitro invasion and in vivo metastases 10, 11. It has also been shown that the tumor invasive edge is highly acidic, and encompasses cells that highly expressed the glucose transporter, GLUT‐1 9.

Therapies targeting tumor microenvironmental acidity using alkalization is becoming widespread. Indeed, it has been shown recently that using a commercially available water alkalizer has an effect in inhibiting melanoma growth 12. We also have documented that neutralizing tumor acidity with oral sodium bicarbonate can lead to a reduction in spontaneous and experimental metastasis in animal models. This therapy does not alter the pH of blood and healthy tissues, and this can be explained by steady‐state physiological reaction–diffusion modeling 13. Sodium bicarbonate is inexpensive and has been shown to be effective in reducing metastases 14. However, at the doses given, the large amounts of sodium are a concern, especially for patients with a history of hypertension.

In this study, we investigate the effect of THAM (trishydroxymethyl aminomethane) on tumor progression and metastasis as an alternative to bicarbonate. THAM, which is also known as Tris–base, is available in the USP and is used clinically in the acute treatment of acidosis, and provided with concentration of 300 mmol/L 15. Unlike bicarbonate, it does not require a counter ion. One clinical study has been performed in ICU patients with mild metabolic acidosis treated with sodium bicarbonate or THAM, which showed that the alkalization effect was similar. In addition, the serum sodium level was decreased after THAM, whereas they were increased after sodium bicarbonate 16.

Our current results demonstrate that chronic ingestion of Tris–base was well tolerated by mice at 200 or 400 mmol/L ad libitum. Neither dose resulted in overt toxicity. A moderate but insignificant increase in granulocytes and hyperkalemia was observed at 400 mmol/L. Further, our results showed that 200 mmol/L THAM was highly effective in inhibiting metastasis in PC3M prostate metastatic model, as well as tumor formation and metastases in a Mia Paca‐2 pancreatic cancer model. We also observed decrease in expression of GLUT‐1 in the treated tumors. Thus, this study supports the use of Tris–base as an alternative to bicarbonate.

## Materials and Methods

### Chemicals and antibodies

Tris (trishydroxymethyl aminomethane) base was obtained from Sigma‐Aldrich, St. Louis, MO, and was dissolved in tap water at a concentration of 200 mmol/L or 400 mmol/L. The pH of the Tris solution was adjusted to 8.4 (using a solution of 1 mol/L HCl) and then given to the mice ad lib. Cell culture media and supplies were obtained from Thermo Fisher Scientific, Carlsbad, CA. D‐Luciferin was obtained from Gold Biotechnology, St. Louis, MO. Polyclonal rabbit anti‐mouse GLUT‐1 antibody was purchased from Millipore (used at concentration of 1:800).

### Experimental design

All animals were maintained in accordance with IACUC standards in the Moffitt Cancer Research Center (Tampa, FL) Vivarium. Established specific pathogen‐free husbandry practices were followed, and twelve‐hour light/dark cycles were applied. All imaging and measurements were performed within the facility. All mice were purchased from Charles River (MA).

For toxicity studies, C3H male mice (5–6 weeks old) were used. These mice were chosen because myelosuppression is a common dose‐limiting toxicity, and these mice are immunocompetent and hence more suitable for safety studies (cf. SCID and Nu/Nu mice) and will facilitate the translation to clinic. Mice were randomly divided in to three cohorts: first cohort was supplied with tap water, the second cohort was supplied with 200 mmol/L Tris–base (pH 8.4), and the third cohort was supplied with 400 mmol/L Tris–base (pH 8.4) for 90 days. For the pancreatic cancer model, female nude mice Nu/Nu (6–8 weeks old) were used, as described previously (ref). For the prostate cancer model, SCID/beige male mice (6–8 weeks old) were used, as per the Caliper (Hopkinton, MA) packet, wherein it was demonstrated a 100% metastatic frequency, compared to 50% frequency in nude mice; 3 days prior to inoculation with tumor cells, mice were randomly divided in to two treatment cohorts: The first cohort was treated with tap water, and the second cohort was treated with 200 mmol/L Tris–base (pH 8.4). To directly compare Tris–base with bicarbonate in an orthotopic pancreatic model, 3 days prior to tumor cell inoculation, mice were randomly divided in to three treatment groups: tap water control, 200 mmol/L bicarbonate, and 200 mmol/L Tris–base. To monitor the water consumption, the weights of the water bottles were recorded before and after providing them to the animals. Animal weights were measured and recorded twice weekly, and the overall health of each animal was noted to ensure timely end points within the experiment.

### Cell culture and inoculation

PC3M cells (‐Luc6 clone) were obtained from Caliper (Hopkinton, MA), and cultured using MEM/EBSS media, supplemented with 10% fetal bovine serum, 1% penicillin–streptomycin, 1% nonessential amino acids, 1% sodium pyruvate, and 1% MEM vitamins. MIA PaCa‐2 pancreatic cells were obtained from American Type Culture Collection, ATCC, Manassas, VA (ATCC), and were cultured in DMEM supplemented with 10% fetal bovine serum and 1% penicillin–streptomycin. All cells were maintained at 37º in 5% CO_2._ Mycoplasma and cell line authenticity testing was completed for all cell lines.

In preparation for inoculation into mice, the cells were trypsinized and rinsed once with sterile phosphate‐buffered saline (PBS) prior to re‐suspension. For experimental metastases, 200 *μ*L containing 5 × 10^6^ cells in PBS was injected directly and slowly (over the course of 1 min) into the tail vein of each mouse and cell distributions were verified by bioluminescent imaging immediately following injection. For orthotopic model, 50 *μ*L containing 1 × 10^6^ cells in PBS was injected into the head of the pancreas. Briefly, the mouse was placed ventral side up and an incision is made midline, 4 mm long. The pancreas was pulled out onto sterile gauze and held in place with a sterile cotton swab. The needle was left inside for a few seconds and then gently retracted. The pancreas was gently placed back inside the abdominal wall and monitored for any leakage or hemorrhaging. The abdominal wall was sutured closed with 5–0 suture, and then, the skin was closed with wound clips. The wound clips were removed 10–14 days postsurgery.

### Urine pH

Urine was obtained by applying gentle pressure on against the mouse abdomen for 10 sec. pH of urine was recorded with pH strips.

### Blood Chemistry, pH, and CBC

The blood pH and electrolytes concentration were obtained using an iSTAT portable clinical Analyzer (Abaxis) with CG8 + cartridges. Blood samples (about 200 *μ*L) were obtained from cardiac stick of the mouse and inserted to the cartridge, and readings were recorded according to manufacturer's specification***.*** Blood CBC was analyzed using ProCyte DX Hematology analyzer (IDEXX Laboratories, Westbrook, ME).

### Bioluminescent imaging

Mice previously inoculated with Luc tumor cells were injected intraperitoneally with 10 *μ*L per g body weight of sterile D‐Luciferin substrate prepared in PBS at 15** **mg/ml (resulting dose 150 *μ*g/g body weights). After 5 min, mice were anesthetized with isoflurane. After 5 min, mice were transferred to the thermo‐regulated, light‐tight chamber of the In Vivo Imaging System, IVIS‐200 (Caliper; Hopkinton, MA). Photographic images were acquired first, and the bioluminescent images were overlaid on top of these images. Bioluminescent images were acquired by measuring photons emitted from luciferase‐expressing cells and transmitted through the tissue. The exposure time for the bioluminescent image acquisition ranged from 0.5 sec (whole tumor images) up to 2 min (lung metastases) to ensure nonsaturation, and differences in exposure time were corrected by expressing data as total flux in photons/sec, rather than photon counts. Images were analyzed using the Living Image software (Caliper; Hopkinton, MA).

### Histology

For subchronic toxicity studies: At necropsy, the brain, the lung/heart, the liver, the spleen, and the kidneys of C3H mice were collected, weighed, and processed for histology.

For other studies: At necropsy, the Lung/Heart, liver, and pancreas were collected. Tissues were processed, embedded in paraffin, and 4‐ to 5‐*μ*m slices of the tissues were obtained. Consecutive sections from each tumor were stained with hematoxylin and eosin (H&E) and/or GLUT‐1 and were graded by a pathologist for presence of tumor tissue. Histology slides were scanned using the Aperio^™^ (Vista, CA) ScanScope XT with a 20x/0.8NA objective lens (200x) at a rate of 2 min per slide via Basler trilinear array.

### Image analysis

An Aperio (Vista, CA) Positive Pixel Count^®^ v9.0 algorithm with the following thresholds: [Hue Value = 0.1; Hue Width = 0.5; Color Saturation Threshold = 0.04; IWP(High) = 220; Iwp(Low) = Ip(High) = 175; Ip(low) = Isp(High) = 100 Isp(Low) = 0] was used to segment positive staining of various intensities. The algorithm was applied to the entire digital image to determine the percentage of positive biomarker staining by applicable area.

### Positive pixel counting

The algorithm was applied to the entire slide's digital image to determine the percentage of positive GLUT‐1 staining by detecting the number of pixels that satisfy the color and intensity specification defined above (GLUT‐1 staining) divided by the number of pixels in nonstained tissue. The quantification of lung and/or liver metastasis was measured using Aperio Genie pattern recognition software, a “teachable” image analysis program, to identify tumor tissue in each section of lung and liver, and to determine the percentage of the section analysis area that was occupied by tumor. The training algorithm developed above was quality‐controlled by a practicing pathologist.

### Magnetic resonance imaging

Tumors MR images were obtained on a Varian MR imaging spectrometer ASR310 (Agilent Life Sciences Technologies, Santa Clara, CA) equipped with nested 205/120/HDS gradient insert and a bore size of 310 mm operating at a magnetic field strength of 7 Tesla. Before imaging, the animals were placed in an induction chamber and anesthetized with 2% isoflurane delivered in 1.5 L/min oxygen ventilation. Upon complete induction, animals were restrained in a custom‐designed holder and inserted into the magnet while constantly receiving isoflurane. Body temperature (36°± 1°C) and respiratory function were monitored continuously (SAII System). A 35‐mm Litzcage coil (Doty Scientific) was used to carry out axial T2‐weighted fast spin‐echo multislice experiments [acquired with TE/TR (echo time/repetition time) = 72 msec/1000 msec, field of view (FOV) = 35 × 35 mm^2^, matrix = 128 × 128, yielding a spatial in‐plane resolution of 273 mm, with slice thickness of 1.5 mm]. Tumor volumes were obtained from these T2‐weighted datasets and measured by manually drawn regions of interest (ROIs) encompassing the entire tumors.

### Tris–base titration curve

First, the initial molar concentration of Tris–base was calculated. At the initial point of the titration curve, pH of the solution was measured with no HCl added, and then, pH was measured after addition of 1 mL of HCl (1 mol/L), to solution. Titration curve was generated by adding increasing amounts of a solution 1 mol/L of HCl to a solution of 0.2 mol/L of Tris (total of 40 mL).

### Statistical analysis

A two‐tailed unpaired student t‐test was employed to determine statistical significance. All survival curves were evaluated by the log‐rank (Mantel–Cox) test and the Gehan–Breslow–Wilcoxon test. A *P* < 0.05 was considered statistically significant. Prism 5 software was used for all statistical calculations.

## Results

### Chronic consumption of 200 mmol/L and 400 mmol/L Tris–base has no toxicity on C3H mice

**Figure 1:**
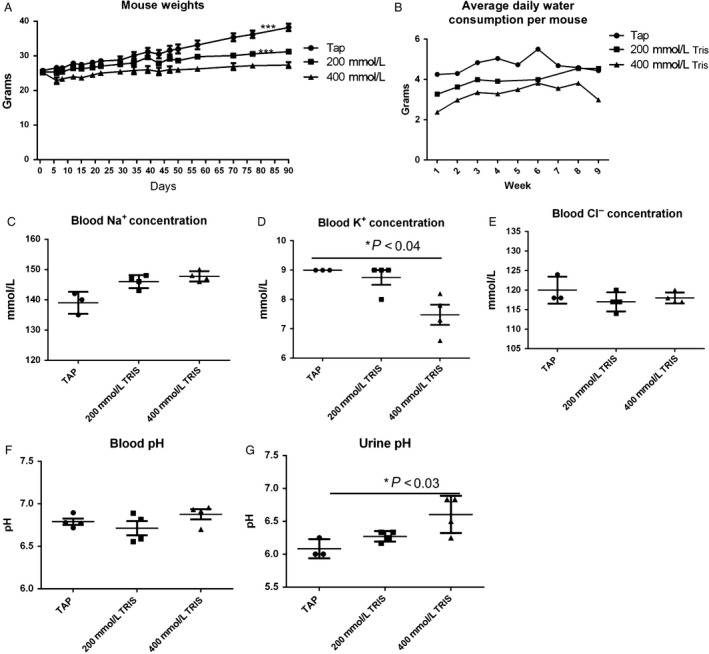
(A) Average mouse weight: animals were weighed throughout the experiment, and the group averages per days were graphed: no significant differences were observed between different groups in the cohort up to 70 days, and significant difference was observed between tap and 400 mmol/L Tris–base (***P = 0.0001) and between 200 mmol/L and 400 mmol/L Tris–base (*P* = 0.0003). (B). Water consumption: water bottles were weighed twice weekly. Water consumption per mouse per day was graphed. (C) Blood sodium concentration. (D) Blood potassium concentration (E). Blood chloride concentration of the three cohorts treatment, tap (*n* = 3), 200 mmol/L, and 400 mmol/L of Tris–base (*n* = 4), was measured using i‐STAT portable clinical Analyzer (Abbott Diagnostics), showing significant increase in K+ between tap and 400 mmol/L Tris–base.(*P < 0.04) (F) Blood pH, (G) Urine pH was measured, and significant differences between tap and 400 mmol/L Tris–Base were observed (*P < 0.03). Mean ± standard error of the mean (SEM) is shown. A two‐tailed Student's *t*‐test was used to calculate statistical significance.

**Figure 2:**
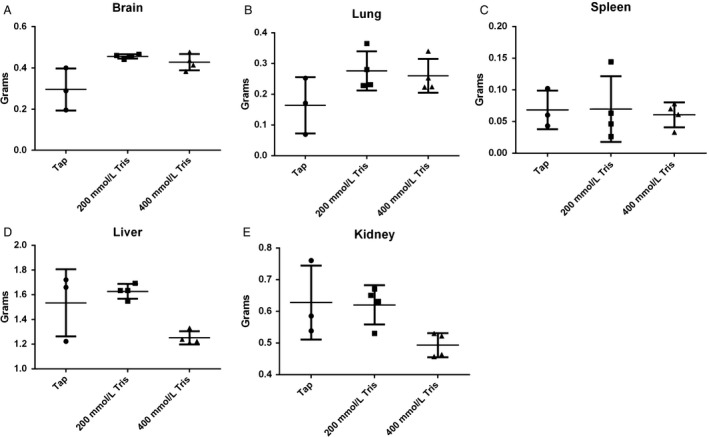
Comparison of organ weights between the treatments groups. Weights of (A) brain, (B) lung, (C) spleen, (D) liver, (E) kidney of the three groups of treatment, tap, 200 mmol/L, and 400 mmol/L of Tris–base. (*n* = 3 for tap and *n* = 4 for treated groups), were measured at necropsy. Nontreated group tend to have smaller brain and lungs and bigger liver and kidney, but the difference was not significant. Mean ± standard error of the mean (SEM) is shown. A two‐tailed Student's *t*‐test was used to calculate statistical significance.

To investigate the toxicity of Tris–base, we placed C3H mice on 200 mmol/L and 400 mmol/L of Tris–base pH 8.4 for 90 days. Mouse weight and water consumption were recorded twice weekly. At the end of the experiment, blood and urine were collected from each mouse to measure their pH, mice were then euthanized, and organs were collected and weighed. All mice survived till the end of 90‐day study, and no abnormalities of conditions or behavior were observed up to 70 days. We observed no significant differences in mouse weights between tap water and 200 mmol/L Tris–base up to 70 days, after that, mice weight started to drop on the 400 mmol/L‐treated mice compared to tap‐ and 200 mmol/L‐treated mice (***P < 0.003) while the 400 mmol/L Tris‐treated group had a slower weight gain in comparison, which may be due to a diuretic effect of Tris–base. Water consumption was different between the treatments, with the control mice consuming more than either of the treated groups **(**Fig. 1A and B**)**. At the end of the experiment, blood Na^+^ and Cl^−^ electrolytes analysis did not differ significantly between treatment groups, but K^+^ levels were significantly lower on the 400 mmol/L Tris–base‐treated group compared to control (*P* < 0.04) (Fig. 1C, D and E). The hematological finding did not show any differences in hemoglobin (Hb) or white blood cell counts. Comparison of white blood cell components between nontreated (tap water) and treated (200 or 400 mmol/L Tris) groups using serial blood sampling of mice showed an increase in granulocytes/lymphocytes ration, in the 400 mmol/L‐treated group which indicates alteration in the immune cells in favor of antitumor (Fig. S2 and S3). Furthermore, no significant differences in blood pH were observed between different groups, consistent with compensated metabolic alkalosis. The increase in urine pH in the 200 mmol/L and 400 mmol/L Tris–base compared to control (*P* < 0.03) confirmed the renal compensation of alkalosis **(**Fig. 1F and G). Weight of brain, lung, spleen, liver, and kidneys were recorded at the end of the experiment and also showed no differences between the treatment groups (Fig. 2A–E). Furthermore, histology of liver, spleen, and lung showed no notable abnormalities (data not shown).

### Response of metastasis to 200 mmol/L Tris–base treatment in prostate model

**Figure 3:**
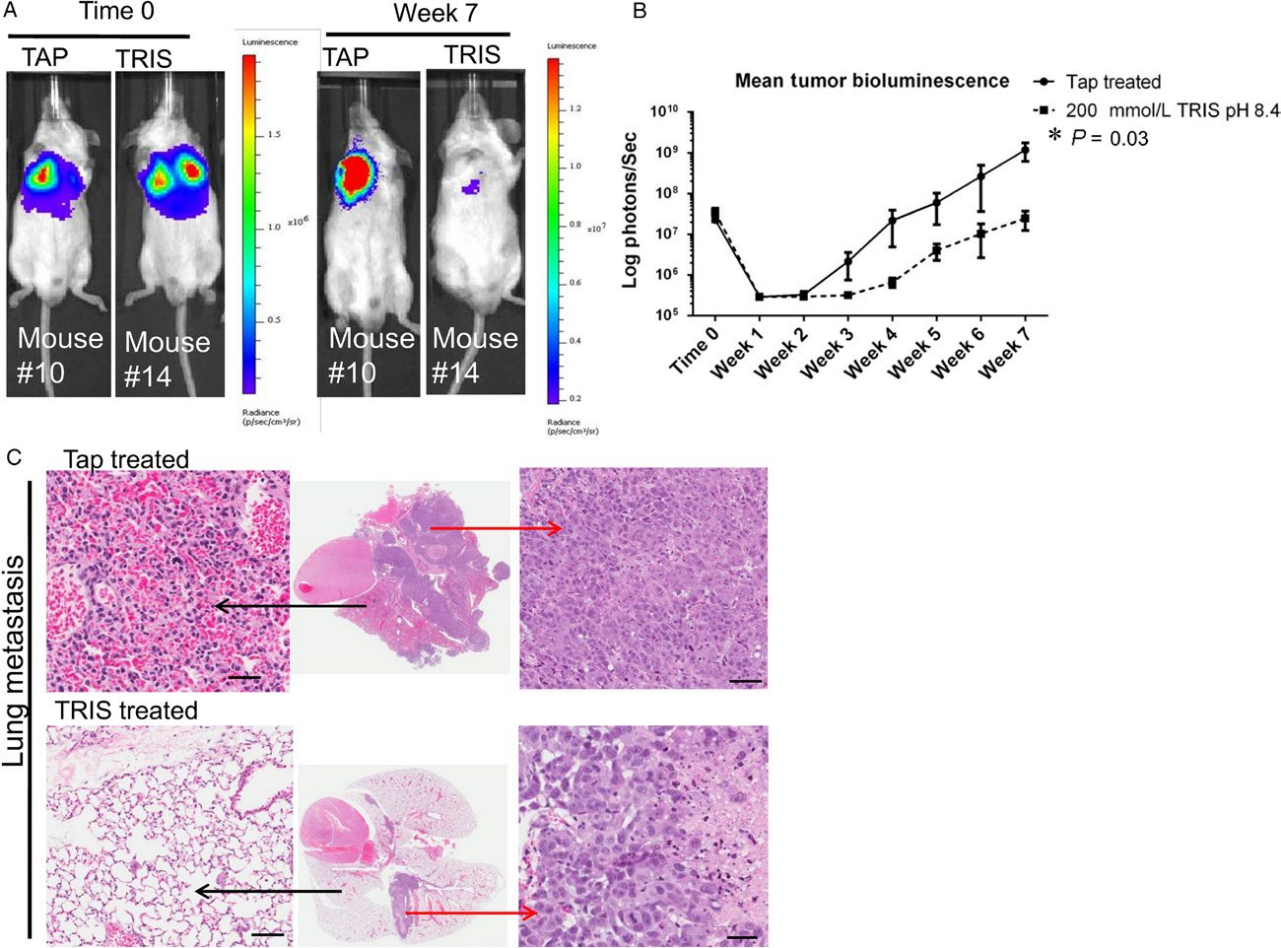
200 mmol/L Tris–base reduces metastasis in prostate cancer model. (A) Representative in vivo bioluminescence images of experimental metastasis model of PC3M‐Luc‐C6 prostate cancer treated with tap and 200 mmol/L Tris–base. Images are at time 0 and week 7, (B) mean tumor bioluminescence in each group after induction of experimental metastases, indicating significantly fewer metastases in 200 mmol/L Tris–base group than in the tap (*P* = 0.03; note log scale), (C) representative H&E images from mice at end point (7 weeks), showing less metastatic lesions in a lung of 200 mmol/L Tris–base‐treated mouse. Black arrow showing normal lung and red arrow showing metastasis. All histological zoomed images are at 200× magnification, and scale bar is 100 *μ*m. A two‐tailed Student's *t*‐test was used to calculate statistical significance.

As 200 mmol/L Tris–base had a less significant effect on body weight and K^+^ compared to 400 mmol/L Tris–base, we tested whether 200 mmol/L Tris–base will reduce metastasis in prostate model using bioluminescent imaging of a human prostate carcinoma cell line, PC‐3M‐luc‐C6, to monitor noninvasively in vivo growth and response of tumors after treatment. Titration curve of 200 mmol/L Tris–base was conducted to confirm the complete neutralization of solution at pH 8.4(Fig. S1). PC3M‐luc‐C6 cells were injected in to the tail vein of male SCID mice; hence, we have shown in numerous prior studies that the intravenous metastatic prostate cancer model is exquisitely sensitive to inhibition with buffer therapy. Mice were then treated with or without 200 mmol/L Tris–base pH 8.4 (*n* = 10 for each group) during 7 weeks. Representative in vivo images of mice of each cohort are shown in Figure 3A, and quantification of bioluminescence signal (measured in mean photon/sec) is shown in Figure 3B; both indicate significant inhibition in metastasis in treated group compared to tap (*P* = 0.03). Reductions in lung metastasis were confirmed by histological examination of tissue (Fig. 3C).

### Response of survival and tumor growth to 200 mmol/L Tris–base (pH 8.4) in pancreatic model

**Figure 4:**
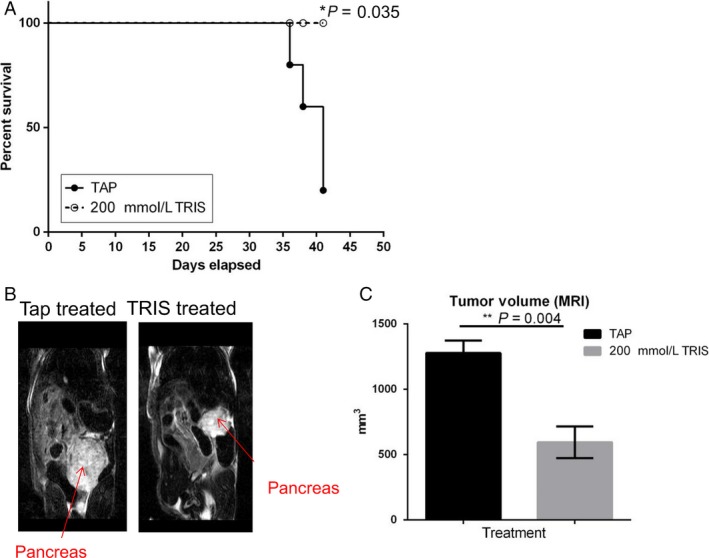
200 mmol/L Tris–base treatment increases survival and decreases tumor formation in Mia PaCa‐2 orthotopic model. (A) Kaplan–Meyer survival curve illustrating that the animals treated with 200 mmol/L Tris–base survived significantly (*P *< 0.035) longer than the nontreated group.(B) Representative coronal T2‐weighted MRI images of the two cohorts, tap water and 200 mmol/L Tris–base (*n* = 5), showing the pancreatic tumor (red arrow). (C) Quantitative assessment of tumor volume at end point, showing a significant decrease in tumor volume in 200 mmol/L Tris–base‐treated group (**P = 0.004).Bar graph represents mean tumor volume ± SEM.

To evaluate in vivo effect of 200 mmol/L Tris–base (pH 8.4) on a model of pancreatic cancer, we injected human Mia PaCa‐2‐luc cell line orthotopically into the pancreata of female nude mice. Mice were then treated with or without Tris–base (*n* = 5) and imaged weekly with bioluminescence imaging (IVIS 200) and with MRI at the end point. Increased survival and significant reductions in primary tumor volume (**P = 0.004) were observed in Tris—base‐treated compared to control (Fig. 4 A, B, and C). To compare the effect of Tris–base to bicarbonate buffer, mice (*n* = 4 each) were treated with tap, 200 mmol/L bicarbonate, or 200 mmol/L Tris–base 3 days after they were inoculated with Mia PaCa‐2‐luc cell line orthotopically. Mice were observed for tumor formation using bioluminescence imaging; 200 mmol/L Tris and 200 mmol/L bicarbonate significantly inhibited the tumor growth with the same efficacy compared to tap (*P < 0.015) (Fig. S4 A, and B).

### Effect of 200 mmol/L Tris–base on tumor aggressiveness and metastasis

**Figure 5:**
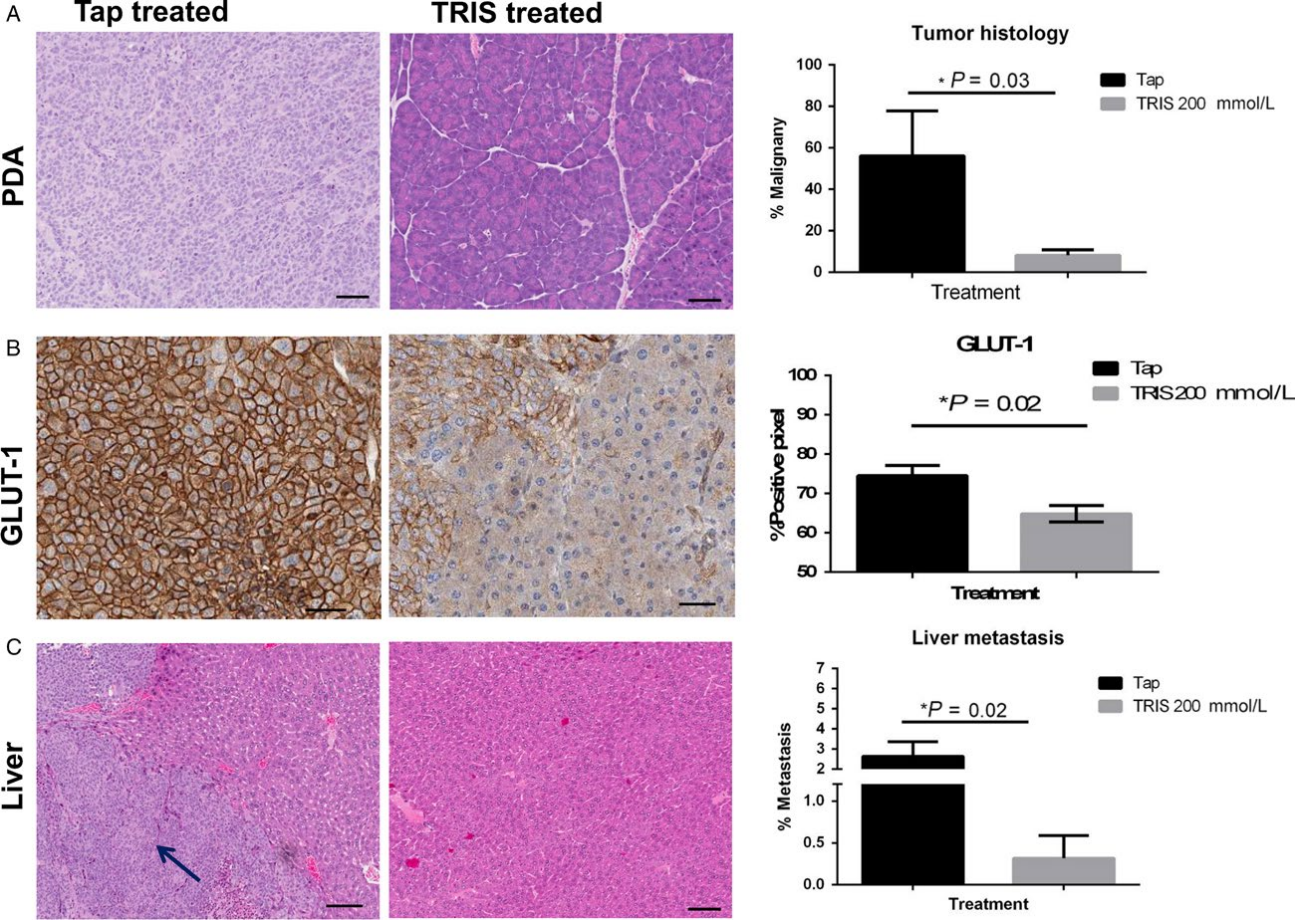
Effect of Tris–base on tumor aggressiveness. (A). Representative H&E staining of pancreas of both tap water and Tris–base‐treated groups (*n* = 5, each group), and quantitative analysis of pancreatic H&E showing percent pixels associated with benign and malignant phenotypes in pancreatic tumors of both treatment cohorts, showing significant higher malignancy in tap group (*P* = 0.03). (B) GLUT‐1 immunohistochemistry stain of pancreas of both treatment groups, showing higher expression of GLUT‐1 in tap cohort, and quantification showing a significant increase in expression in tap, in comparison with 200 mmol/L Tris–base‐treated (*P* = 0.02). (C) Liver metastasis in the two treatment cohorts, and quantification (percentage of liver section occupied by tumor measured by (area of tumor/total lung area)*100), showing significantly lower metastasis in treated group (*P = 0.02). Mean ± standard error of the mean (SEM) is shown.

It is well known that malignant tumors express enhanced glucose metabolism, and there is a correlation between GLUT‐1 expression level and the grade of tumor aggressiveness 17. To evaluate this, first we quantified the percent of malignancy in pancreatic tumor in the two cohorts, showing that those mice treated with Tris–base decreased significantly the percent of malignancy compared to the nontreated (*P* = 0.03) (Fig. 5A). Further, GLUT‐1 expression was analyzed and was significantly lower in the treated group (*P* = 0.02) (Fig. 5B). To further evaluate the correlation of GLUT‐1 and metastasis, we quantified liver metastases, in this orthotopic model which is the most frequent metastatic site for pancreatic cancer. These analyses showed a significant reduction of metastasis in treated group compared to control (Fig. 5C).

## Discussion

Our results are consistent with previously published model predictions that alternative buffers can be as effective as bicarbonate in inhibiting tumor progression and metastasis 18. We previously showed that chronic ingestion of 200 mmol/L solution of an imidazole buffer, IEPA, or free‐base lysine reduced metastases in a PC3M mouse models. Notably, the lysine was inhibitory only if ingested in the free‐base form (pH 10.4) and not as the chloride salt (pH 8.4). Even so, all buffers investigated to date contain at least one halide counter ion. Our choice of Tris–base was based on its buffering capacity as well as safety in clinic. Tris–base is known in clinic as THAM; tromethamine, with a pH adjusted to 8.4 is injected intravenously for the correction of metabolic acidosis. It acts as a proton acceptor and prevents and/or corrects acidosis by actively binding hydrogen ions (H^+^). It binds not only cations of fixed or metabolic acids but also hydrogen ions of carbonic acid, thus increasing bicarbonate anion (HCO3−).

We have shown in this study that mice can well tolerate 200 mmol/L ad lib Tris–base. In addition, our work is also certainly demonstrating the ability of 200 mmol/L Tris–base to inhibit metastasis, which was clearly observed in both prostate and pancreatic preclinical models.

The reduction in GLUT‐1 expression was an unexpected finding. Although increased GLUT‐1 expression is associated with pancreatic cancer aggressiveness, and invasiveness 17, 19, and treatment with Tris reduced aggressiveness, we fully expected that GLUT‐1 would be elevated as a response to compensate for the increase in buffering and acid neutralization. The reduced expression could be a direct result of tumor pH or, given the long‐term treatment, may represent a pH‐dependent clonal selection for a less aggressive phenotype. Such a phenomenon has also been observed in TRAMP models of prostate cancer (Ibrahim‐Hashim et al., Can Res, in review).

In conclusion, it is known that there is a substantial adverse relationship between the acidic tumor microenvironment and cancer progression. This acidity is advantageous for tumor invasion and metastases. The findings presented here show how neutralizing this tumor acidity, by the administration of 200 mmol/L of Tris–base buffer, is sufficient to inhibit tumor progression and metastasis.

## Conflict of Interest

All authors have no conflict of interests to disclose.

## Supporting information


**Figure S1**. (A) The chemical structure of Tris‐base. (B) Titration curve of 200mM Tris‐base.

**Figure S2**. Hematological analysis of blood in three treatment cohorts, Tap, 200mM and 400mM Tris, showing no differences in (A) concentration of Hemoglobin, and (B) white blood cell count.

**Figure S3**. Quantification of white blood cell components on a serial blood sampling in 4 time points for three treatment cohorts, Tap, 200mM and 400mM Tris. (A) Lymphocyte, (B) Monocyte and (C) Granulocytes. (D) granulocytes/lymphocytes ratio on 4 different time points .E. granulocytes/lymphocytes ratio after 21 days of treatment showing significant decrease in the ratio (*p=0.01)

**Figure S4**. Effect bicarbonate and TRIS on misPACA cell line (A) representative bioluminescene images for the three treatment cohorts (TAP, 200 mM bicarboate and 200 mM TRIS).
