# Supplementary figures and images for: Tris–base buffer: a promising new inhibitor for cancer progression and metastasis
Source: Cancer Med. 2017 May 29;6(7):1720–9. doi: 10.1002/cam4.1032 (PMC5504318; doi:10.1002/cam4.1032)

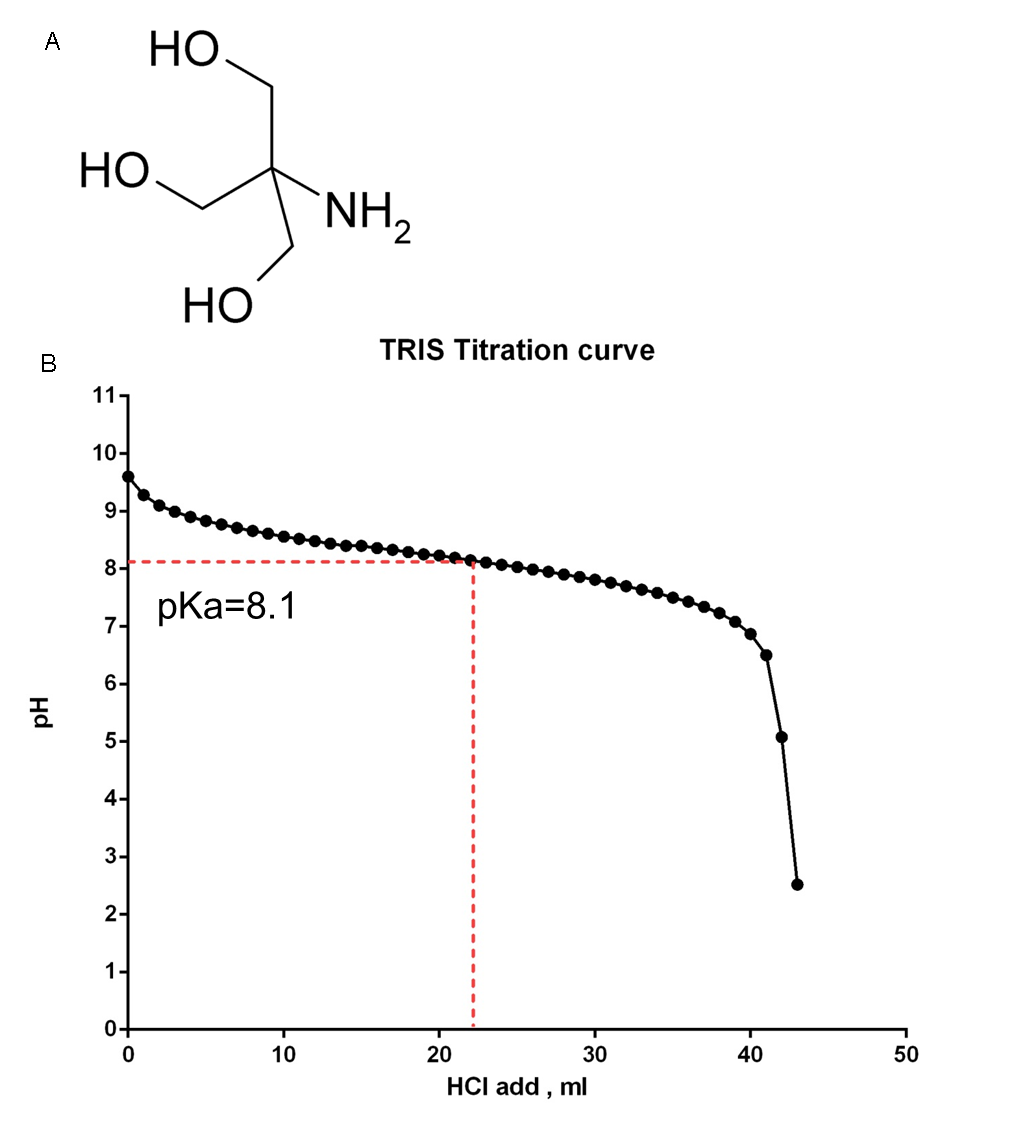

Supplement: Supplementary file 1 — Figure S1. (A) The chemical structure of Tris‐base. (B) Titration curve of 200mM Tris‐base. [file CAM4-6-1720-s001.tif]

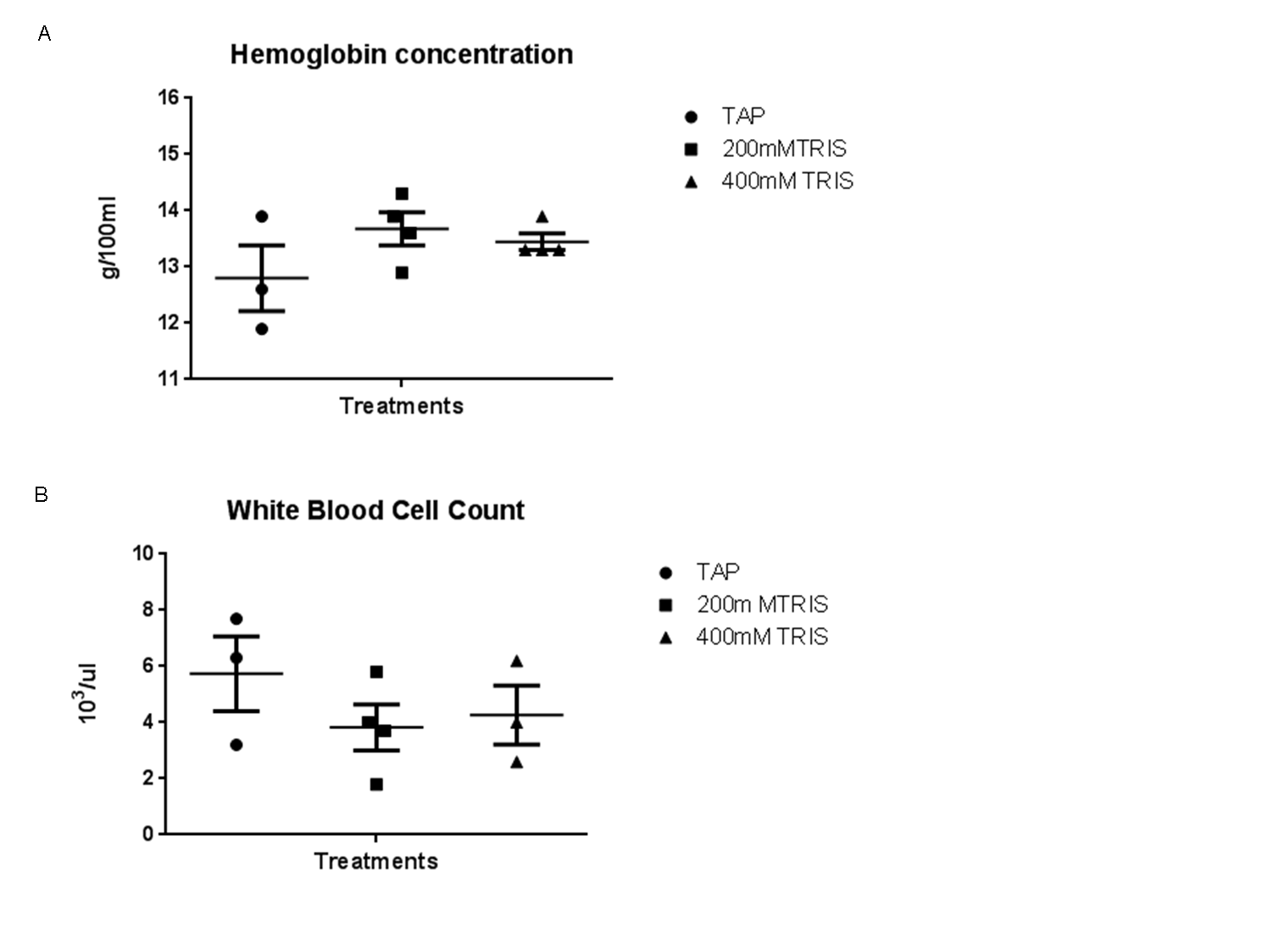

Supplement: Supplementary file 2 — Figure S2. Hematological analysis of blood in three treatment cohorts, Tap, 200mM and 400mM Tris, showing no differences in (A) concentration of Hemoglobin, and (B) white blood cell count. [file CAM4-6-1720-s002.tif]

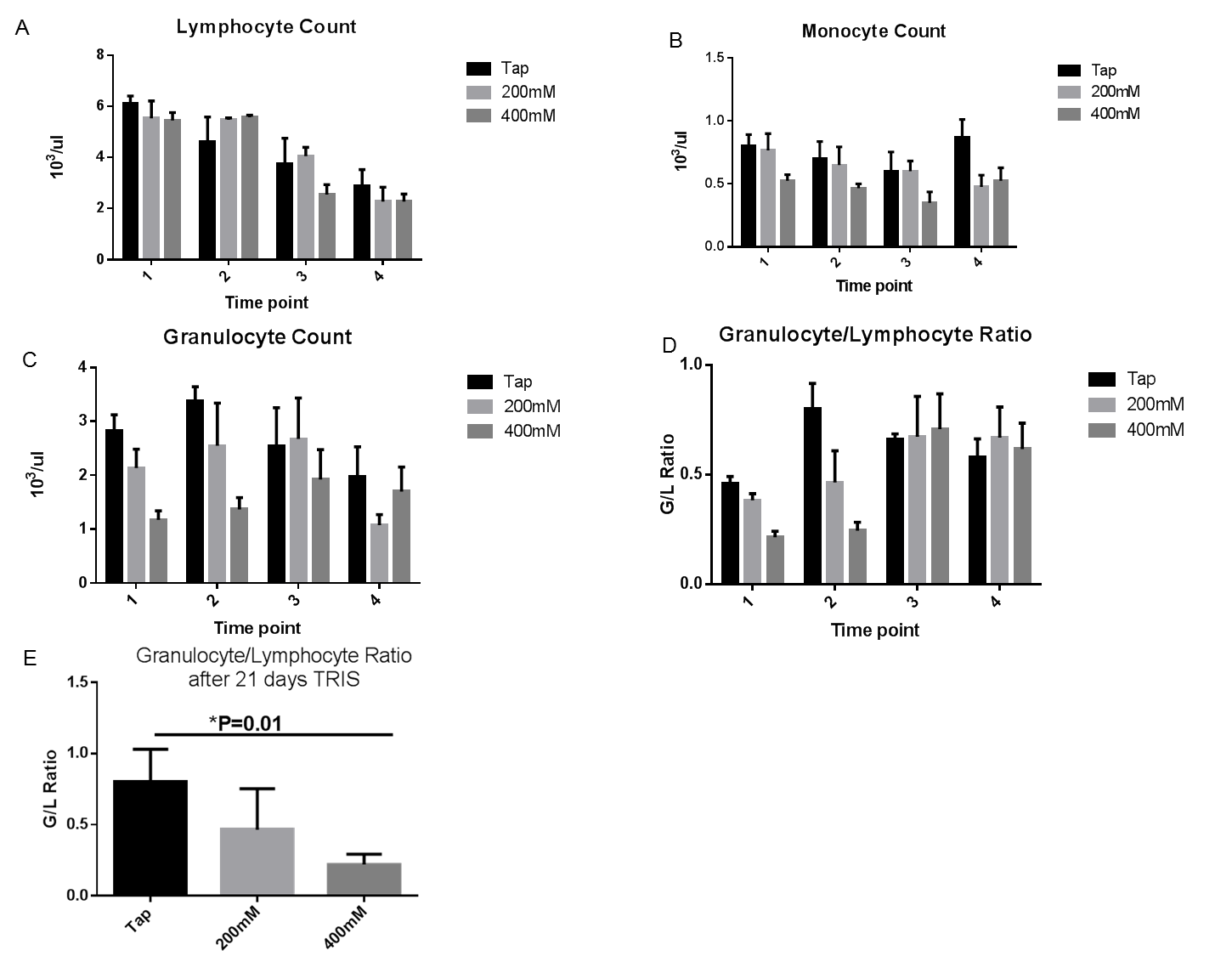

Supplement: Supplementary file 3 — Figure S3. Quantification of white blood cell components on a serial blood sampling in 4 time points for three treatment cohorts, Tap, 200mM and 400mM Tris. (A) Lymphocyte, (B) Monocyte and (C) Granulocytes. (D) granulocytes/lymphocytes ratio on 4 different time points .E. granulocytes/lymphocytes ratio after 21 days of treatment showing significant decrease in the ratio (*p=0.01) [file CAM4-6-1720-s003.tif]

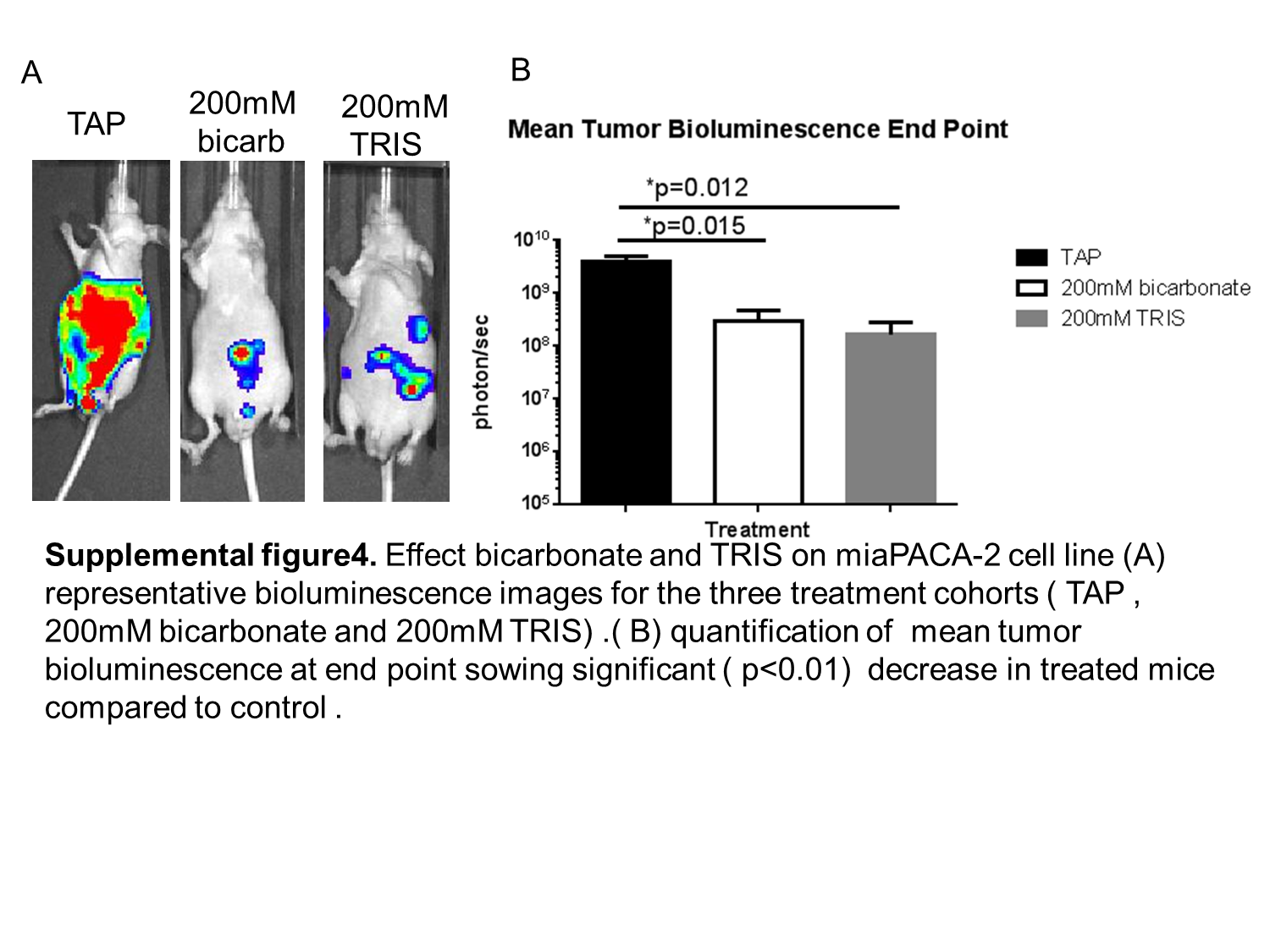

Supplement: Supplementary file 4 — Figure S4. Effect bicarbonate and TRIS on misPACA cell line (A) representative bioluminescene images for the three treatment cohorts (TAP, 200 mM bicarboate and 200 mM TRIS). [file CAM4-6-1720-s004.tif]
